# P-1646. Assessing Antibiotic Adverse Drug Events from Walk-In Clinics at an Academic Healthcare System

**DOI:** 10.1093/ofid/ofae631.1812

**Published:** 2025-01-29

**Authors:** Nishant Patel, Michael Zou, Milner Staub, Emily W Nardone, Retha B Thomas, Angela Zuill, Claude E Shackelford, Jim Zhang, Sharon Onguti

**Affiliations:** University of Louisville School of Medicine; Vanderbilt University, Nashville, Tennessee; Vanderbilt University Medical Center, VA Tennessee Valley Healthcare System, Nashville, TN; Vanderbilt University Medical Center, Nashville, Tennessee; Vanderbilt University Medical Center, Nashville, Tennessee; Vanderbilt, College Grove, Tennessee; Vanderbilt University Medical Center, Nashville, Tennessee; Vanderbilt University Medical Center, Nashville, Tennessee; Vanderbilt University Medical Center

## Abstract

**Background:**

Outpatient antibiotic adverse drug event (ADE) rates are largely unknown. This project aimed to evaluate methods for collecting data on antibiotic ADEs and report antibiotic ADE data from 8 walk-in clinics (WICs) at Vanderbilt University Medical Center (VUMC), which represents a high-volume antibiotic prescribing outpatient setting.

**Figure:**
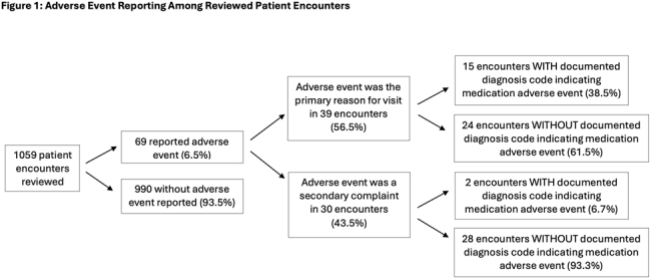

**Methods:**

We attempted retrospective data collection of antibiotic ADE billing codes. After multiple trials, we recognized a need to understand how ADEs are documented (Tables 1 and 2) versus billed. To examine this, we conducted a retrospective chart review of patients prescribed antibiotics in selected WICs from 8/1/23 - 10/31/23 with WIC, primary care, or emergency department visits within 14 days after. We reviewed visit notes for antibiotic ADEs. If present, visit billing codes were collected and analyzed. To evaluate potential new antibiotic ADE data collection methods, especially ADEs reported via calls without a visit, we conducted prospective collection of antibiotic ADE calls or return visits within 14 days from patients prescribed antibiotics at selected WICs from 11/1/23-4/30/24 via voluntary REDCap survey reports by WIC providers. We used manual chart review to assess antibiotic appropriateness.

**Figure:**
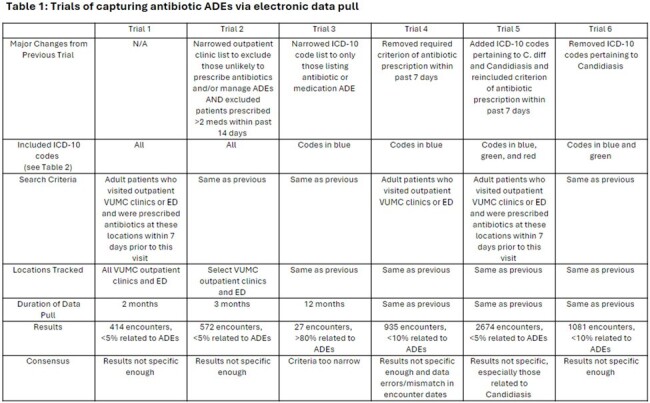

**Results:**

From 8/1/23-10/31/23, we extracted 1059 unique visits with antibiotic prescriptions; 69 (6.5%) had antibiotic ADEs documented in subsequent visits. ADEs were secondary complaints in 30/69 (43.5%) visits. ADE billing codes were present in 17/69 (24.6%) visits (Figure 1).

From 11/1/2023-4/30/2024, 16,274 WIC visits had antibiotic prescriptions. Providers logged 45 calls and 3 return visits (48/16274, 0.3%); 6/48 (12.5%) antibiotics were unnecessary. When antibiotics were indicated, choice, dose, or duration was inappropriate in 13/42 (31.0%).

**Table 2:**
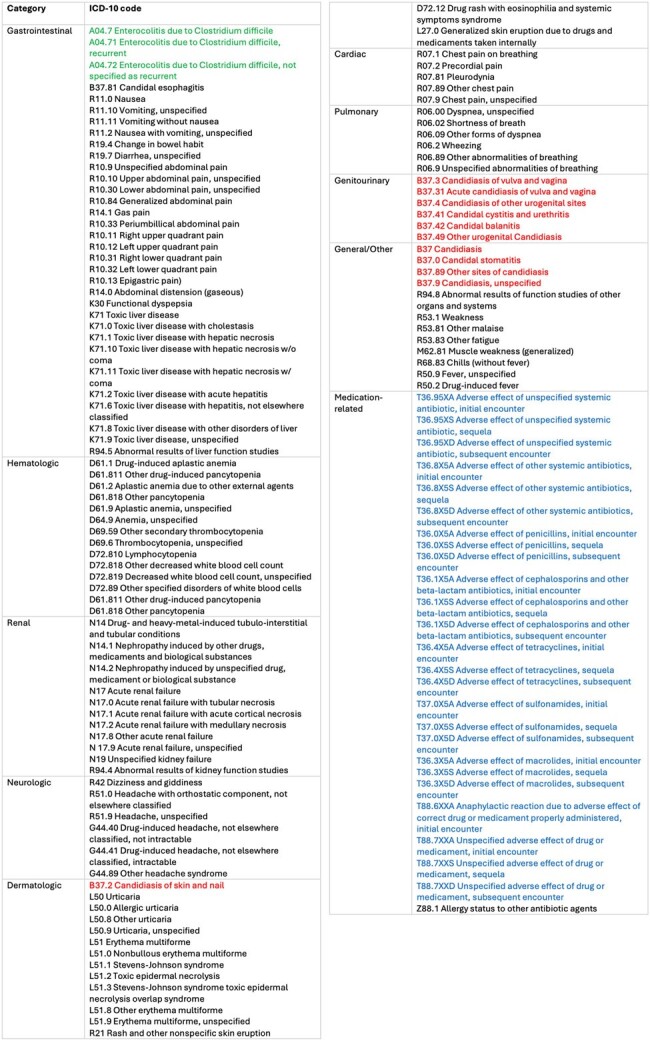
ICD-10 codes classified by organ system

**Conclusion:**

This project showed outpatient antibiotic ADEs occur routinely. Utilizing voluntary reporting led to increased capture of antibiotic ADE patient calls but was not comprehensive. Additionally, ADE billing codes are currently underutilized, resulting in inaccurate estimates of antibiotic ADEs. Future studies are needed to determine an optimal approach to identify and extract antibiotic ADE data from electronic medical records.

**Disclosures:**

**Milner Staub, MD, MPH**, Gilead: Stocks/Bonds (Public Company)|Johnson & Johnson: Stocks/Bonds (Public Company)

